# Gout, Hyperuricemia, and the Intestinal Microbiome

**DOI:** 10.1007/s10753-025-02337-x

**Published:** 2025-07-05

**Authors:** Nicholas Renton, Michael H. Pillinger, Michael Toprover

**Affiliations:** 1 Division of Rheumatology, NYU Grossman School of Medicine, New York, NY USA; 2 Rheumatology Section, VA New York Harbor Health Care System, NY Campus, New York, NY USA; 3 NYU Langone Orthopedic Hospital, 301 E 17th Street, Suite 1410, New York, NY 10003 USA

**Keywords:** Gout, Human microbiome, Prebiotics, Probiotics, Butyrate

## Abstract

Gout is a disease of hyperuricemia (HU) leading to monosodium urate crystal deposition in the joint, resulting in inflammation and joint damage. Recently, efforts have been made to characterize the intestinal microbiome of patients who suffer from HU and gout, and pre-clinical studies have evaluated the utility of prebiotics and probiotics in alleviating gout. Herein we review recent notable studies addressing these topics. In brief, the “gouty” microbiome is characterized by reduced diversity, an elevated *Bacteroides: Firmicutes* ratio, and reduced presence of *Akkermansia* and *Bifidobacterium*. In anserine models, supplementation with *Lactobacillus* probiotic strains appears to reduce serum urate (SU) and HU-induced inflammation. Murine models suggest that the chicory-derived prebiotic inulin may reduce SU, and oral supplementation with the anti-inflammatory short-chain fatty acid butyrate may lower SU by enhancing urate excretion and alleviate HU-induced tissue inflammation. Many of these studies are limited by modest numbers of participants and/or incompletely documented experimental controls, and, in the case of animal models, questionable reproducibility in humans. Many studies have been geographically limited. There remains a need for more information regarding the features of the “gouty” microbiome in wider populations, as well as for additional well-controlled probiotic and prebiotic studies in more physiologically relevant animal models prior to clinical trials.

## Introduction

Gout is a crystal arthropathy driven by hyperuricemia (HU) and affecting 5.1% of the US population [1]. Gout classically causes acute mono- or, less commonly, polyarticular arthritis in response to precipitated monosodium urate crystals in joints. Poorly treated, gout can progress to severe arthropathy (tophaceous gout), potentially leading to disfigurement, ulceration, joint erosion and localized infection. Notably, not everyone with HU develops gout; asymptomatic hyperuricemia (AH), in which serum urate (SU) concentrations exceed the crystal solubility threshold without signs or symptoms, is present in nearly 20% of the US adult population [2]. The factors predisposing an individual with AH to develop gout remain an ongoing area of research.

Collectively referred to as the intestinal microbiome, the trillions of bacteria that live in the human gut modulate human health. Recent studies have examined the role the intestinal microbiome plays in crystal arthropathies [3, 4]. Specifically, both animal and human studies have addressed the role of the microbiome in the development of HU and gout [4, 5]. Liu et al. found that members of the human gut microbiota anaerobically degrade uric acid and reduce SU (potentially compensating in part for the evolutionary loss of human uricase), a function that is abrogated by anaerobe-selective antibiotics [6]. Corroborating this finding, Kasahara et al. (2023) reported that germ-free mice colonized with anaerobic purine-degrading bacteria had significantly lower levels of SU than germ-free mice colonized with bacteria lacking purine-degrading genes [7]. Additionally, Martínez-Nava et al. used a meta-transcriptomic approach to demonstrate that genes involved in pyruvate and pentose phosphate metabolism are over-expressed by the gut microbiome of gout patients in relation to that of AH and normouricemic patients, potentially modulating acetate production and urate metabolism [8]. In addition to characterizing the gouty microbiome and transcriptome, some research has addressed the potential role of probiotics in gout. Yamanaka et al. reported that the *Lactobacillus gasseri* strain PA-3, which is found in some yogurt, can degrade purines and thereby reduce SU levels in a double-blind, placebo-controlled probiotic trial [9]. Thus, microbiome research in gout remains an evolving field. Consequently, there is a need to aggregate and critically appraise the literature in this area.

Herein, we review recent studies addressing the microbiome’s influence on gout pathogenesis as well as its potential therapeutic role. To complete this narrative analysis, we used PubMed and EmBase as literature search engines to review a broad range of recently published papers (2020 or later) spanning various study designs that address the role of the microbiome in HU and/or gout. Search terms included gout, hyperuricemia, microbiome, and microbiota. Searches were carried out between October 2024 and March 2025.

## Bacterial populations in human samples: comparing patients with AH or gout to healthy controls (Table 1)

**Table 1:** Changes in human gut microbiota in hyperuricemia (HUA) and gout

Population	Experimental Group	Control	Increased compared with Controls	Decreased compared with Controls	Authors
307 fecal samples collected from male patients of Guangzhou University of Chinese Medicine, Guangdong Province, China	− 102 male acute gout patients between 15 and 69 years of age	86 male age-matched controls	- *Bacteroidetes: Firmicutes (B: F)* ratio -Phylum: *Bacteroidetes* -Genera: *Prevotella*,* Fusobacterium*	-Overall diversity -Phylum: *Proteobacteria* -SCFA producers: *Roseburia*,* Coprococcus*,* Eubacterium*,* Faecilibacterium prausnitzii* *-*Urate degrading *Enterobacteriaceae*: *Escherichia*,* Klebsiella*,* Enterobacter*,* Citrobacter*	Chu, Huang, et al., 2017 ^10^
Fecal samples from 40 male patients enrolled at Gangnam Severance Hospital in Seoul, Korea	− 14 male acute gout patients before urate-lowering therapy (ULT) AND 9 of those same patients after 30-day ULT − 18 male patients with chronic gout	8 male patients with asymptomatic HU	-*B: F* ratio *reduced by 30-day ULT -*Prevotella copri* *-Enterococcus faecalis*	*-*Overall diversity *-Prevotella: Bacteroides* (P: B) ratio -*Lactobacillus (plantarum*,* fermentum*,* reuteri)* -*Bifidobacterium (bifidum*,* breve*,* catenulatum)* *-Escherichia coli* *-Coprococcus*	Kim, Yoon, et al., 2022 ^12^
Recruited in rural-residing adults over the age of 50 from Hunan Province, China	− 479 patients with HU	1,393 age-and-sex matched healthy controls	NA	-Overall diversity -*Roseburia*,* Ruminococcus*,* Collinsella* -*Coprococcus* *low abundance correlated with higher SU	Wei, Zhang, et al., 2022 ^14^

Using 307 human samples, Chu et al. compared the microbial composition of gout patients having flares to healthy, age-matched controls. The microbiomes of gout patients had lower microbial diversity and richness and an elevated ratio of *Bacteroidetes: Firmicutes (B: F)* [10], similar to prior reports in patients with inflammatory bowel disease [11]. At the phylum level, the relative abundance of *Bacteroidetes* was increased in gout patients while *Proteobacteria* was decreased. Genera overrepresented in gout patients included species from *Prevotella* and *Fusobacterium.* By contrast, healthy controls had greater *Roseburia*,* Coprococcus*,* Eubacterium*, and *Faecalibacterium prausnitzii;* these microbes are known for producing butyrate, a short-chain fatty acid (SCFA) thought to confer anti-inflammatory benefits (see below) [5]. Interestingly, healthy controls also had greater representation of urate-degrading bacteria from the *Enterobacteriaceae* family, including those from the *Escherichia*,* Klebsiella*,* Enterobacter*, and *Citrobacter* genera, suggesting the possibility of direct regulation of urate levels by the gut. The above data were controlled for BMI, as gout patients had significantly higher BMI than healthy controls. Age, alcohol use, probiotic use, and vegetarianism did not differ between gout patients and controls. The authors did not report differences in other relevant co-morbidities such as CKD diagnosis or diabetes mellitus.

HU is a precondition for gout, but not all patients with HU develop clinical disease. Kim et al. examined the gut microbiota of AH male patients to evaluate for possible protective signatures against symptomatic disease [12]. They compared the microbiome of eight patients with AH to three groups of gout patients: (1) 14 patients having gout flare, (2) the same patients after 14 days of treatment with urate-lowering therapy (ULT), and (3) 18 patients with gout in the intercritical period. Consistent with the observations by Chu et al., patients with AH had a lower *B: F* ratio than those with gout diagnoses. Interestingly, treatment of gout with ULT decreased the *B: F* ratio, suggesting restoration of a non-gout microbial ecology. Patients with AH also had lower abundance of *Prevotella copri* and a lower ratio of *Prevotella: Bacteroides* (genus-level) than those in the gout cohorts, potentially corroborating Chu et al.’s findings of increased *Prevotella* in gout patients. Of note, in these analyses gout patients overall had a higher prevalence of CKD compared with those with AH, and the group of patients with chronic gout (on greater than 6 months of urate-lowering therapy) had a higher prevalence of CKD than gout patients assessed during active flares. Other relevant comorbidities including diabetes mellitus, hypertension, dyslipidemia, and alcohol use did not vary between the experimental groups.

Wei and Zhang used cross-sectional data collected from adults over age 50 to compare HU patients to those with normal levels of SU, whether the patients met classification criteria for gout or not [13]. The discovery cohort consisted of 239 HU patients and 1,153 controls. Subsequently, 240 HU patients with 240 age- and sex-matched controls recruited from the Step Study served as the validation cohort [14]. In both cohorts, after adjusting for covariates (age, sex, BMI, substance use, diabetes mellitus, and hypertension), patients with HU had less microbial diversity than controls. Specifically depleted genera in HU patients of the discovery cohort included *Coprococcus* of the *Lachnospiraceae* family, recapitulated in the validation cohort.

In sum, currently available data suggest that the microbiome of patients with both HU and gout are reduced in diversity relative to patients with normal levels of SU, but the effect is more pronounced in patients with gout. Thus, gout may be marked by perturbations in the often-cited *B: F* ratio: two studies in different populations yielded elevated *B: F* ratio in gout patients, although the comparison group was healthy controls in one study and asymptomatic HU patients in the other. Whether this perturbed ratio plays a role in, or at least correlates with, symptomatology rather than HU itself, merits further study. Microbiomes of patients with gout seem to be characterized by *Prevotella*, whereas *Coprococcus* was shown to be more abundant in microbiomes of healthy patients and inversely correlated with SU.

**Fig. 1:**
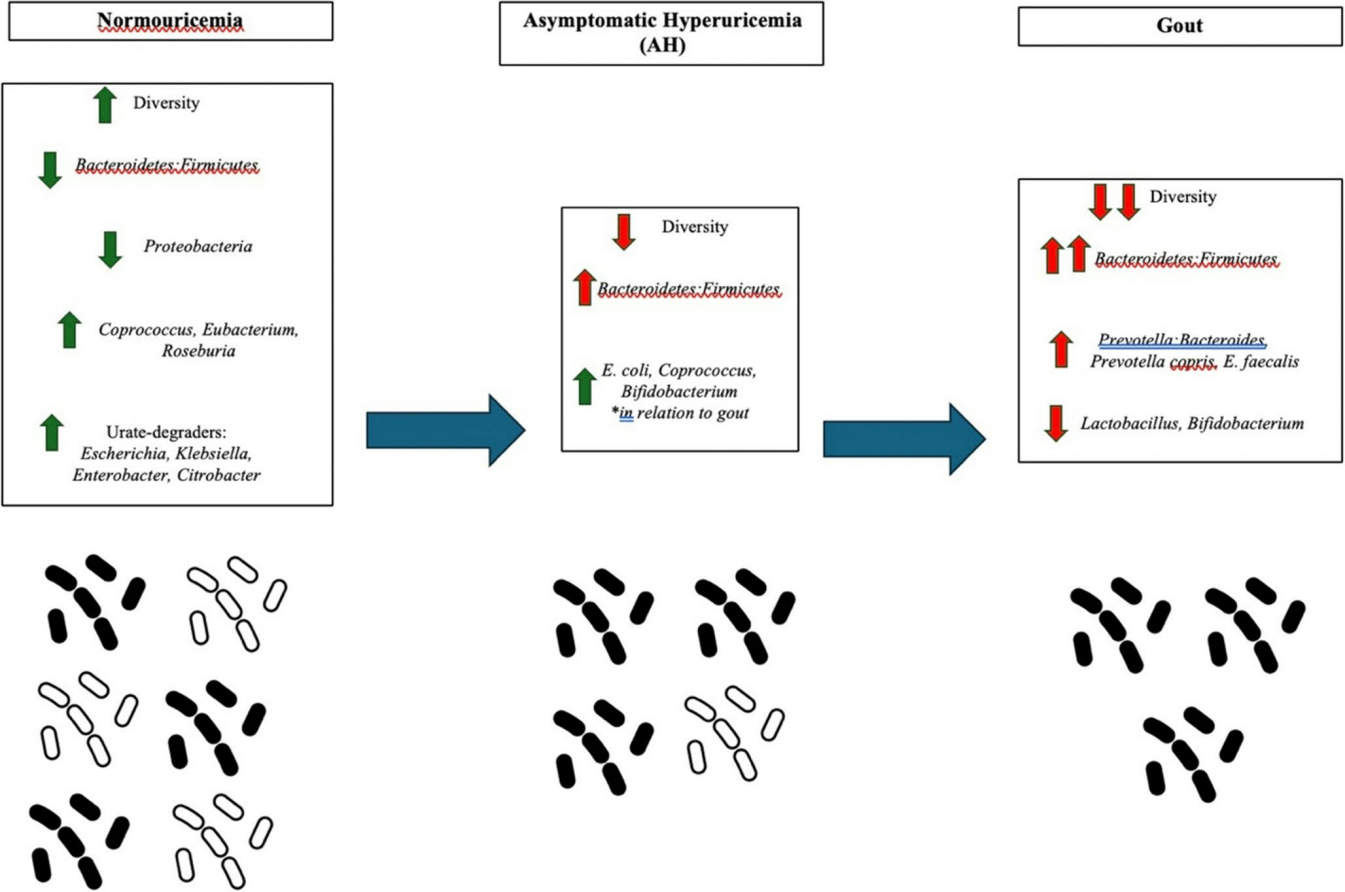
Schematic of different microbiome compositions between normouricemia, asymptomatic hyperuricemia (AH), and gout. Existing data suggest that in humans the microbiome in HU is characterized by significantly reduced diversity and an increased Bacteroidetes: Firmicutes ratio. Among individuals with gout, reductions in Lactobacillus and Bifidobacterium have additionally been noted. In relation to individuals with HU, individuals with normal levels of urate have greater Coprococcus, Roseburia, and Eubacterium as well as purported urate-degrading bacteria from Escherichia, Klebsiella, Enterobacter, and Citrobacter

## Animal Studies: Probiotic Supplementation and Effects on Urate Levels and Gout Models (Table 2)

**Table 2:** Summary of animal model (murine, anserine) studies of Microbiome manipulation in hyperuricemia (HUA)

Animal	Mode of Hyperuricemia Induction	Intervention	Control	Result of Intervention in comparison with Control	Authors
Male Magang goslings	High-calcium high protein diet (HCP)	*Lactobacillus rhamnosus* GG (LGG) or LGG + metabolites gavage x 14 days	PBS gavage x 14 days	- Reduced SU, BUN, Cr, xanthine oxidase (XOD) - Reduced pro-inflammatory cytokines (IFN-γ, IL-1β, TNF-α)	Fu, Chen, et al., 2024 ^15^
		*Lactobacillus plantarum* SQ001 gavage or *L. plantarum* SQ001 + metabolites x 14 days	PBS gavage x 14 days	- Reduced SU, BUN, Cr, XOD - Reduced pro-inflammatory cytokines (IFN-γ, IL-1β, TNF-α) - Increased *Lactobacillus*,* Butyricicoccus*,* Ruminococcus* in fecal microbiome	Fu, Luo, et al., 2024 ^17^
Male C57BL/6 mice	Uricase knockout (KO)	Standard chow + Inulin supplementation via gavage x 49 days	Standard chow	- Reduced SU - Reduced pro-inflammatory cytokines (IL-6, IL-1β, TNF-α) and LPS - Increased fecal SCFA (butyrate, acetate, propionate) - Increased microbial diversity - Increased *Ruminococcus*,* Akkermansia*,* Bifidobacterium*	Guo, Yu, et al., 2021 ^18^
Male C57BL/6 mice of different ages: young (~ 3 months), old (~ 18 months), and aged (~ 24 months)	MSU injection into the hind paw and/or peritoneum	Butyrate 200 mg/kg via gavage x 14 days	No gavage	Young mice: - Reduced foot thickness in young mice - Reduced pro-inflammatory cytokines in peritoneum, foot, and serum (IL-1β, TNF-α, IL-6) Older/aged mice: - Reduced SU - Reduced GLUT9 (renal urate re-absorption protein) - Increased ABCG2 (colonic urate-excretion transporter)	Song, Gao, et al., 2024 ^21^

Unlike humans who lack uricase, most mammals degrade urate enzymatically. Consequently, researchers have employed alternative animal models to study HU and gout [15]. For example, geese lack uricase and develop both HU and symptoms—joint effusion and swelling, MSU crystal accumulation in tissues—resembling human gout. Fu et al. fed day-old male goslings a high calcium and protein (HCP) diet to induce HU [15]. Like HU-humans, HU-geese had reduced microbial abundance, with specific reductions in the family *Lachnospiraceae*,* Ruminococcus*,* Butyricicoccaceae* and increased *Collinsella* and *Desulfovibrionales* (which has been linked to metabolic syndrome in mouse models) [16]. To the authors’ surprise, the “healthy” bacteria *Lactobacillus* and *Lactobacillus rhamnosus* GG (LGG), which have some evidence of probiotic potential in animal and human disease, also experienced relative increases in the HU geese in relation to controls, which the authors suggest may serve a protective role.

Fu et al. then examined the effect of supplementation with the *Lactobacillus rhamnosus* strain LGG on HU. Four experimental groups were used: (1) control goslings, (2) HU geese, (3) HU geese also given LGG gavage (> 1*10^9^ CFU), and (4) HU geese given gavage with LGG along with its supernatant metabolites collected in vitro. HU geese given LGG and LGG + metabolites experienced increased *Lactobacillus*, *Butyricicoccaceae* and *Ruminococcus* expression in relation to HU-only geese. LGG or LGG + metabolite supplementation also resulted in significantly reduced SU, Cr, BUN, and xanthine oxidase (XOD), IFN-$\:\gamma\:$, and IL-1ß, although other markers of gout (e.g. knee thickness) were not evaluated.

Fu, Luo et al. conducted a similar experiment with a different strain of Lactobacillus, *L. planatrum* SQ001 [17]. Again, HU goslings experienced significantly elevated SU, XOD, BUN, Cr, with SU exceeding the threshold for symptomatic disease (420$\:\mu\:$mol/L). HCP goslings had poorer growth and greater mortality (~ 40% of HU geese at 28 days vs. <2% in the control group), suggesting severe metabolic disease from the HCP diet al.one. Changes in gut microbiota in the HU group were similar to those reported in Fu et al.’s preceding work described above. HU goslings given *L. plantarum* SQ001 and *L. plantarum* SQ001 + metabolites (harvested from supernatant in vitro) via oral gavage experienced improved weight and decreased mortality in relation to the other HCP goslings, and reductions in UA, XOD, BUN, and Cr as well as levels of IL-1ß, TNF-$\:\alpha\:$, and INF-$\:\gamma\:$. The phylum *Firmicutes* families *Lachnospiraceae*,* Ruminococcaceae*,* Lactobacillaceae*, and *Butyricicoccaceae*, were increased in HU goslings given *L. plantarum* SQ001, which the authors believed to signify reversal of HCP-diet-induced dysbiosis in geese. Future studies would benefit from more specific evaluation of the microbial metabolites of *Lactobacilis sp* and their effects.

## Prebiotic Influence on HU and Gout

Prebiotics are non-digestible food elements that theoretically nurture the growth of beneficial gut bacteria. Guo et al. investigated the role of the prebiotic inulin in alleviating HU [18]. Inulin is reported to alter gut populations and may also serve as a substrate for generation of short-chain fatty acids (SCFAs), which have been reported to decrease gut inflammation [5]. In this case, the authors developed a male C57BL/6 mouse uricase knockout (KO) model capable of developing HU [19]. They established three experimental groups of eight mice each: WT mice fed standard chow, KO mice fed standard chow, and KO + I mice fed standard chow plus inulin supplementation via oral gavage. After seven weeks, the KO group had significantly increased SU (9.51 ± 0.46 mg/dl) in relation to WT mice (2.55 ± 0.33 mg/dl), suggesting successful HU establishment. Mice in the KO + I group, intriguingly, had mean SU 6.39 ± 0.20 mg/dl, representing a 30% reduction from the non-inulin KO group. KO + I mice also had lower levels of inflammatory cytokines compared to KO mice. KO + I mice had significantly increased fecal SCFAs including acetate, propionate, and butyrate compared to KO mice.

Guo et al. further evaluated the effect of inulin supplementation on microbial community structure and function. Compared to controls, KO mice demonstrated a significantly elevated *B: F* ratio. KO mice also had reduced abundance of the butyrate-producing bacteria *Akkermansia* and *Ruminococcus.* The elevated *B: F* ratio was not restored by inulin gavage in the KO + I group, but inulin supplementation did increase the abundance of *Akkermansia* and *Ruminococcus* as well as *Bifidobacterium* (also butyrate- producing). KO mice had lower diversity than WT mice, while inulin supplementation significantly increased diversity in relation to KO mice not receiving the probiotic.

Finally, Guo et al. correlated levels of specific metabolic markers with changes in microbial composition. *Ruminococcus* was positively correlated with tight junction protein ZO-1 expression and negatively with IL-1 β, IL-6, LPS, and, most importantly, SU. *Akkermansia* was positively correlated with fecal acetate and butyrate.

In summary, mouse and anserine models of hyperuricemia show altered microbiota, favoring certain species, however, the relevance to humans is yet to be determined.

## Effects of Butyrate and Other SCFAs in Animal Models of Hyperuricemia and Gout

In vitro studies have shown that butyrate can suppress monosodium urate (MSU) crystal-induced inflammation [20]. Song et al. recently employed a murine model to examine the intestinal microbiome and the impact of butyrate on gout outcomes across the lifespan [21]. Male C57BL/6 mice divided into three age groups—young (~ 3 months), old (~ 18 months), and aged (~ 24 months)–received subcutaneous injections of monosodium urate (MSU) crystals into the dorsal hind paw to induce a gout flare phenotype. Upon injection of MSU crystals, old mice experienced greater tissue inflammation (i.e., foot thickness) than young mice. Both old and aged mice had greater tissue levels of IL-1ß and SU, which the authors interpreted as greater sensitivity to MSU-induced inflammation.

These initial assays were followed by a series of cross-age fecal microbiota transplant (FMT) experiments: each group had gut microbials harvested on day zero, received a broad-spectrum antibiotic cocktail for three days, and then underwent two rounds of FMT with microbiota from a different age group (or phosphate buffered saline (PBS) control), at which juncture the microbiota were reassessed prior to MSU crystal injection into the hind paw. Young mice transplanted with FMT from old or aged mice exhibited increased footpad swelling in response to MSU crystal injection compared with the PBS control and had elevated pro-inflammatory cytokines–along with significantly increased SU. In contrast, while old or aged mice receiving FMT from young mice had reduced SU as well as lower pro-inflammatory cytokine signaling in relation to controls, they did not experience reduced tissue inflammation (foot thickness).

Microbiome analysis after cross-aged FMT showed shifts in microbial composition, although the *B: F* ratios did not change. Young mice given FMT from old or aged mice had reduced measures of diversity post-transplant, suggesting a decline in microbial diversity with aging. Specifically, *Bifidobacterium* declined when young mice were given old or aged FMT, while *Lachnoclostridium* within *Lachnospiraceae* increased in old or aged mice given FMT from young mice. *Akkermansia* was a dominant species in young mice and butyrate metabolism was enriched in young mice as well as old/aged mice given young microbiome via FMT. Taken together, the authors concluded that *Bifidobacterium* and *Akkermansia* must produce butyrate, which may be critical in controlling gout. Indeed, higher levels of butyrate (as well as acetic acid and propionic acid) were found in young mouse fecal pellets in relation to aged mice, although they do not report results from old mice.

The authors proceeded to develop a butyrate supplementation model, in which mice in all three age groups (but not receiving cross-age FMT) received 14 days of butyrate via oral gavage before MSU crystal injection into both the hind-paw and peritoneum. Butyrate-supplemented young mice had reduced foot thickness and pro-inflammatory cytokines in foot tissue, as well as in peritoneal fluid and serum, compared to non-butyrate supplementation. While neither foot thickness nor pro-inflammatory cytokines were addressed in older and aged mice given butyrate, butyrate supplementation in these mice did reduce SU. The authors therefore proposed that butyrate supplementation in the gut may enhance renal and colonic uric acid excretion. Indeed, butyrate-supplemented aged mice showed reduced expression of GLUT9 (urate re-absorption protein in kidney) and increased expression of ABCG2 (colonic urate-excretion transporter). The authors also showed increased mRNA for ZO-1, occludin, and junctional adhesion molecule A in the colons of mice both old and aged mice; these genes contribute to intestinal barrier integrity via tight junctions.

In sum, aging mice are predisposed to HU-induced disease, which was correlated with reduced *Akkermansia* and *Bifidobacterium* and lower levels of fecal butyrate. Mice receiving direct butyrate supplementation had decreased foot thickness, pro-inflammatory cytokine signaling, and lower SU, although due to sparse documentation in the methods it is unclear whether the comparison group was adequately controlled (e.g., via PBS or sham gavage). Consequently, the role of butyrate in modulating gout symptoms, particularly microbial-derived butyrate, deserves further study.

**Fig. 2:**
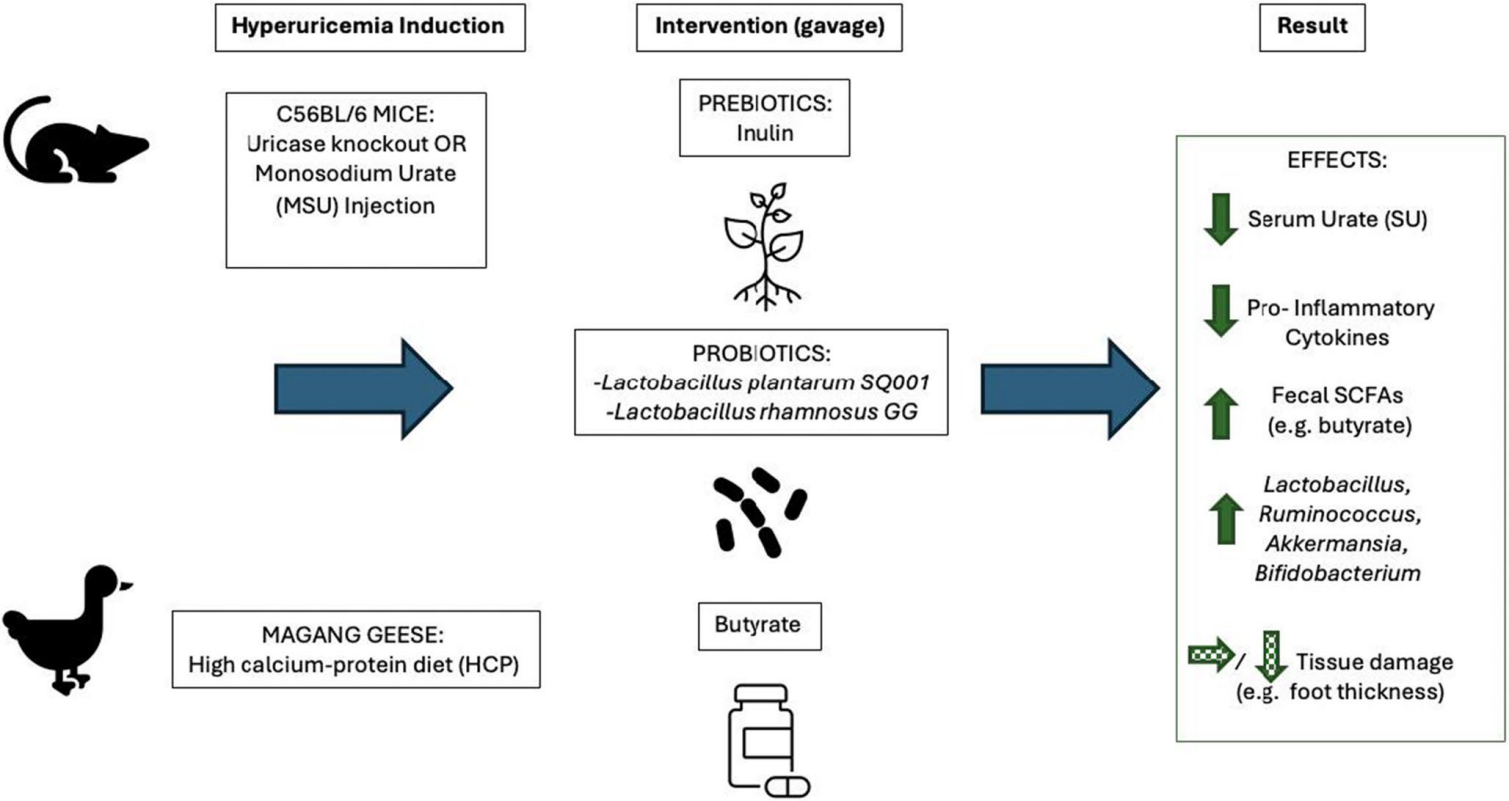
Schematic of microbiome modulation approaches to alleviate hyperuricemia (HU) in animal models. Reviewed animal models induce HU in mice via uricase knockout or injection of monosodium urate (MSU) crystals or in geese via diet. After HU is established, the intervention of interest (prebiotic, probiotics, or butyrate) is provided to the animal via gavage. Collectively, animal models receiving the intervention have shown reduced serum urate, lower levels of pro-inflammatory cytokines, increases in fecal SCFA comment, and greater abundance of certain bacterial populations in relation to controls. Only some of these models report that the intervention reduces HU-induced tissue damage

## Discussion

This review highlights recent active areas of microbiome research in HU and gout. Studies on human feces in cohorts of > 300 patients in Guangzhou, China and 40 patients in Seoul, Korea, demonstrated elevations in the *Bacteroidetes: Firmicutes* (*B: F*) ratios in gout patients [10, 12]. This phylum-level ratio is often cited in the microbiome literature as a marker of gut homeostasis, with perturbations correlating with dysbiosis and various disease states [11]. Interestingly, both obesity and metabolic syndrome are characterized by a decreased *B: F* ratio [22], whereas gout appears to be associated with an increased *B: F* ratio. Of note, bacteria within the phylum *Firmicutes* are dominant producers of the short-chain fatty acid butyrate, which is purportedly anti-inflammatory, anti-obesogenic, and anti-carcinogenic [23]. In addition to the elevated *B: F* ratio, gout patients may have overrepresentation of bacteria from the genus *Prevotella*, including *P. copri* (a potential risk factor for rheumatoid arthritis) and increased *Prevotella: Bacteroides* ratios [24]. Ultimately, reduced microbial diversity appears to be a hallmark of the “gouty” microbiome, although it remains unclear whether such a microbiome is a contributor to or consequence of the disease of gout. Future studies would benefit from more consistent comparison groups to promote confidence in the stability of more granular findings—e.g.,* Firmicutes* are reduced in gout– and recruiting patients with other ethnic backgrounds and countries of origin. Nonetheless, current work on the human “gouty” microbiome sets the stage for studies examining if and how the microbiome could be manipulated via prebiotics and probiotics to modulate SU levels and/or alleviate gout symptoms, most of which to date have taken place in animal models.

As noted above, research evaluating potential probiotic strains for HU/gout alleviation has been carried out in geese, principally in two separate studies by Fu and collaborators [15, 17]. Fu et al. showed that both *Lactobacillus rhamnosus GG* (LGG) and *Lactobacillus plantarum* SQ001 could alleviate gout in geese fed a HU-inducing diet. In both studies, HU mice given LGG or *L. plantarum* SQ001 via gavage experienced greater reductions in SU, pro-inflammatory cytokine levels and markers of kidney injury than control HU mice gavaged with PBS, although the authors did not confirm these observations with other probiotic strain(s), or strains which would be considered “unhealthy” for alleviating HU as direct comparison. The authors also did not report on changes in joint thickness, a physical parameter of gout burden they reported elsewhere in the manuscript. Notably, their HU model induced severe metabolic disease, resulting in death in many of the HU geese, Thus, while the probiotic studies are intriguing, the lack of direct comparison with other candidate strains and the extreme nature of HU-induction for experimental purposes suggests that different animal models are needed for further validation of these products. Furthermore, it is not clear whether *L. plantarum* SQ001, isolated from the geese upon which the authors experimented, is a viable probiotic in humans.

Prebiotics have also received some attention as therapies for gout. Guo et al. studied the effect of the inulin in male uricase-knockout mice, reporting a 30% reduction in SU in mice receiving inulin in relation to control mice, as well as a reduction in some pro-inflammatory cytokines and an increase in fecal SCFA content [18]. Guo et al. unfortunately did not comment on any changes in renal function or other end-organ damage after inulin supplementation. They did note that uricase-KO mice supplemented with inulin had increased diversity and abundance of butyrate-producing bacteria *Akkermansia* and *Ruminococcus*. While these results are intriguing, in the absence of data concerning HU-induced end-organ damage, it is difficult to obtain a precise picture of whether inulin would confer benefits in gout symptoms. The SCFA butyrate, a down-regulator of inflammation in other inflammatory diseases, was evaluated as a direct supplement by Song et al. using a mouse model [21]. They found reduced inflammation (tissue thickness in response to MSU as well as pro-inflammatory cytokines) and SU in some groups of butyrate-supplemented mice.

This narrative review raises several important limitations. First and foremost, the literature remains limited; our review includes only seven reports. Aside from the reported metabolic studies, publications looking at microbial functions are extremely limited. Furthermore, all seven studies included in this review were conducted in East Asia, potentially introducing bias in both human and animal models. For instance, the features of the microbiome associated with gout patients in Korea and two regions of China– Guangzhou and Hunan Province– may differ from those of geographically diverse populations, and immune proclivities (e.g., HLA types) may be distinct. Thus, there is a need for similar trials in other populations, particularly with human data from patients of varying ethnicities and nationalities. The relevance of animal models of HU and gout to human disease remains difficult to interpret; uricase knockout mice tend to handle HU worse than humans, who probably have compensatory mutations to manage the high-urate environment, and birds are evolutionarily very distinct.

In sum, the microbiome of patients with gout appears to differ from that of healthy adults with normal levels of SU. Dominant features include an elevated *B: F* ratio, reduced gut diversity, and reduced presence of butyrate producers. There are noteworthy results in anserine and murine experiments regarding the potential of a butyrate-producing and/or urate-lowering strains to lower SU, specifically from the *Lactobacillus* genus, although many of these studies suffer from overall modest numbers of experimental replicates, less-than-robust controls and/or direct comparison groups, and the artificial nature of HU induction. Butyrate, however, does appear to have potential benefits on HU and gout, whether as a product of probiotic strains or when provided as a direct supplement. Butyrate’s effects merit further study in gout patients, particularly since oral supplementation of sodium butyrate has been shown to be feasible and potentially beneficial in other human metabolic and inflammatory conditions [25].

## Data Availability

No datasets were generated or analysed during the current study.
